# SNX3-retromer requires an evolutionary conserved MON2:DOPEY2:ATP9A complex to mediate Wntless sorting and Wnt secretion

**DOI:** 10.1038/s41467-018-06114-3

**Published:** 2018-09-13

**Authors:** Ian J. McGough, Reinoud E. A. de Groot, Adam P. Jellett, Marco C. Betist, Katherine C. Varandas, Chris M. Danson, Kate J. Heesom, Hendrik C. Korswagen, Peter J. Cullen

**Affiliations:** 1 School of Biochemistry, University of Bristol, Bristol, BS8 1TD UK; 2 Hubrecht Institute, Royal Netherlands Academy of Arts and Sciences and University Medical Center Utrecht, Uppsalalaan 8, Utrecht, 3584 CT The Netherlands; 3 Program in Cell Biology, University of California, San Francisco, 16th Street, San Francisco, CA 94158 USA; 4 Proteomics Facility, School of Biochemistry, University of Bristol, Bristol, BS8 1TD UK; 5 Present Address: The Francis Crick Institute, 1 Midland Rd, London, NW1 1AT UK; 6 Present Address: Laboratory of Developmental Genetics, The Rockefeller University, 1230 York Avenue, New York, NY 10065 USA

## Abstract

Wntless transports Wnt morphogens to the cell surface and is required for Wnt secretion and morphogenic gradients formation. Recycling of endocytosed Wntless requires the sorting nexin-3 (SNX3)-retromer-dependent endosome-to-Golgi transport pathway. Here we demonstrate the essential role of SNX3-retromer assembly for Wntless transport and report that SNX3 associates with an evolutionary conserved endosome-associated membrane re-modelling complex composed of MON2, DOPEY2 and the putative aminophospholipid translocase, ATP9A. In vivo suppression of *Ce-mon-2*, *Ce-pad-1* or *Ce-tat-5* (respective MON2, DOPEY2 and ATP9A orthologues) phenocopy a loss of SNX3-retromer function, leading to enhanced lysosomal degradation of Wntless and a Wnt phenotype. Perturbed Wnt signalling is also observed upon overexpression of an ATPase-inhibited TAT-5(E246Q) mutant, suggesting a role for phospholipid flippase activity during SNX3-retromer-mediated Wntless sorting. Together, these findings provide in vitro and in vivo mechanistic details to describe SNX3-retromer-mediated transport during Wnt secretion and the formation of Wnt-morphogenic gradients.

## Abstract (web summary)

Sustained Wnt secretion requires the endosomal SNX3-retromer complex for endosome-to-trans-Golgi network transport of the internalised Wnt chaperone Wntless. Here the authors show that in both *C. elegans* and human cells, SNX3-retromer requires an evolutionary conserved membrane remodelling complex for Wntless sorting and Wnt secretion.

## Introduction

Wnts are a highly conserved family of acylated and glycosylated secretory proteins, which control many aspects of development through the regulation of diverse processes such as cell proliferation, cell fate determination and migration^1,2^. Wntless (Wls) transports Wnt morphogens from the endoplasmic reticulum through the Golgi and on to the cell surface and, in some tissues, may also play a role in apical to basolateral transcytosis for polarised Wnt secretion^3,4^. Wls is thus indispensable for Wnt secretion and the establishment of Wnt morphogenic gradients across higher metazoans^5–7^.

Upon reaching the plasma membrane, the fate of Wls differs from that of the secreted Wnt. Wls is internalised in an adaptor protein-2 (AP2) and clathrin-dependent manner^8–10^. Subsequently, it is recognised and retrieved from the early endosome back to the Golgi by the sorting nexin-3 (SNX3) containing retromer complex (SNX3-retromer)^9,11–15^. By retrieving Wls, this pathway prevents its lysosomal degradation and allows Wls to undergo COPI-mediated retrograde transport back to the endoplasmic reticulum, where it can assist further rounds of Wnt secretion^16^.

The SNX3-retromer mediates Wls retrieval independently of the SNX-BAR (sorting nexins with Bin, Amphiphysin, Rvs (BAR) domain)-containing retromer (SNX-BAR-retromer)^14,15^. The SNX-BAR-retromer is composed of two distinct multiprotein assemblies^17,18^. The first is a hetero-trimer of VPS26 (VPS26A and VPS26B in humans), VPS29 and VPS35, herein termed retromer^19^. Retromer functions as an endosome-associated recruitment hub for both cargo and associated accessory proteins^20^. A hetero-dimer of SNX-BAR proteins, SNX1 or SNX2, complexed to either SNX5, SNX6 or SNX32, forms a second protein assembly which is responsible for membrane deformation and tubular carrier formation^21–24^. It is postulated that the SNX-BAR proteins generate membrane curvature through the insertion of an amphipathic helix into the lipid bilayer, resulting in the generation of positive membrane curvature, stabilised by the concave-shaped BAR domain and the formation of higher-ordered helical assemblies^25–27^.

SNX3 associates with the VPS35:VPS26:VPS29 retromer through direct binding to VPS35, but does so independently of the SNX-BAR proteins, to form the SNX3-retromer^14,28–30^. The SNX3-retromer also associates with Wls through two Φ-X-[L/M] motifs in the Wls cytoplasmic tail to mediate cargo capture^15,31,32^. To promote its subsequent recycling, the capture of Wls must be coupled to membrane deformation and carrier formation. Here, we elucidate the central importance of SNX3 binding to retromer for Wnt secretion and, by employing quantitative proteomics combined with in vitro biochemical and in vivo genetic analyses in *C. elegans*, we reveal that an evolutionary conserved complex containing a putative aminophospholipid translocase (flippase) is required for SNX3-retromer-mediated trafficking of Wls and Wnt secretion.

## Results

### Mapping of the SNX3 interaction with VPS35

**Fig. 1:**
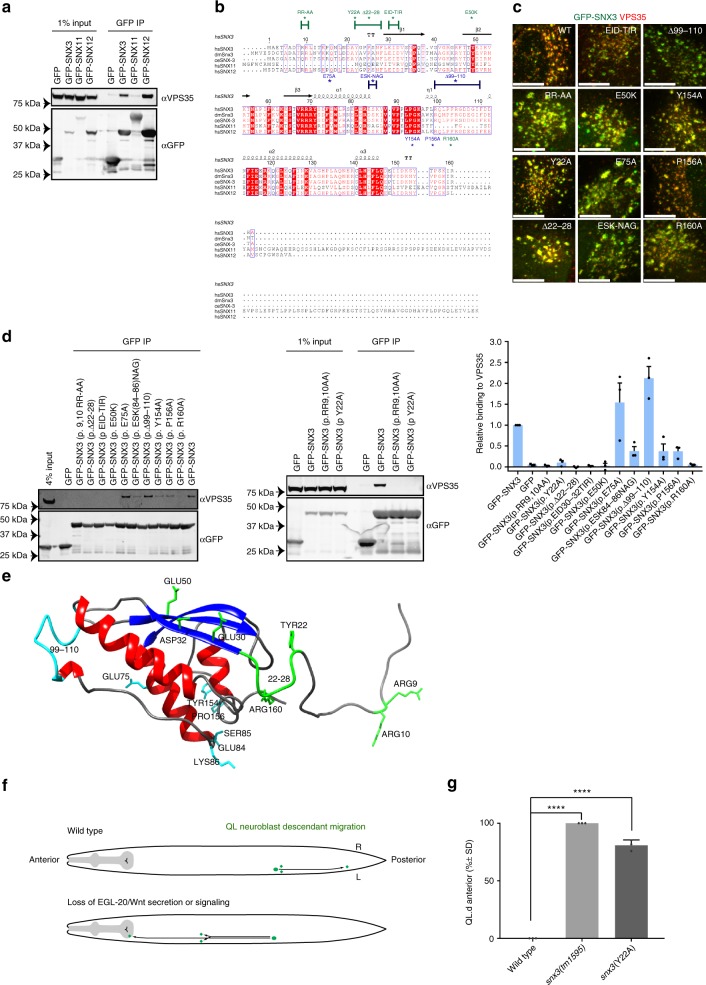
The association of SNX3 with VPS35 is essential for the endosomal sorting of Wls. **a** HEK293T cells were transiently transfected with GFP-tagged SNX3, SNX11 and SNX12. The lysates were subjected to a GFP-trap immuno-isolation and immuno-blotted with the indicated antibodies. **b** Sequence alignment of human SNX11, SNX12, SNX3 and SNX3 homologues using ESPript 3.0 (76). The residues and regions mutated in the present study are highlighted. **c** Merged confocal images of HeLa cells transiently transfected to express wild-type or mutated versions of GFP-SNX3 (green) and immuno-stained for endogenous VPS35 (red). The scale bar indicates 11.5 μm (see Supplementary Fig. 1 for the entire panel of images). **d** HEK293T cells were transiently transfected to express the indicated GFP-SNX3 construct, subjected to a GFP-nanotrap and immuno-blotted with the indicated antibodies. The fluorescent bands, from three independent biological replicates, were quantified using a Licor Odyssey Scanner to show the relative binding of the GFP-SNX3 constructs to VPS35. Error bars indicate s.e.m. **e** Mapping of the targeted mutations onto the NMR derived structure of full length SNX3 (33). Green residues show those mutants that lead to a loss of VPS35 association while those residues in blue have limited effects on VPS35 binding when mutated. Note that the extended amino-terminal region prior to the β1-strand is intrinsically disordered (33). **f** Schematic representation of the migration of the QL neuroblast descendants (QL.d) and the dependency of this migration on Wnt signalling. In wild type, the QL.d migrate to positions in the posterior part of the animal. In the absence of Wnt signalling, the QL.d migrate in the opposite, anterior direction. **g** Mutation of SNX-3 p.Y22A induces a defect in QL.d migration (the cells migrate towards the anterior instead of the posterior). ***p < 0.0001 (Student’s *t*-test from three independent biological replicates; error bars indicate s.d.). Note that complete loss of snx-3 function induces a fully penetrant defect in QL.d migration (14)

Previously we, and others, have shown that SNX3 directly associates with the VPS35 retromer subunit^14,15,29,30,32^ (Fig. 1a), and that both SNX3 and retromer are required for the in vivo endosome sorting of Wls^14,15^. To establish the functional importance of the SNX3 interaction with retromer, and as a prelude to more detailed mechanistic analysis of the SNX3-retromer sorting pathway, we performed a targeted mutagenic screen to identify SNX3 mutants that lacked the ability to associate with VPS35.

Like other PX domains, SNX3 comprises a three-stranded anti-parallel β-sheet (β1 through to β3) followed by a four-helical bundle^33^. Both the amino-termini and carboxy-termini are intrinsically disordered^34^. Sequence alignments were carried out to compare SNX3 from across higher metazoans together with human SNX12 and SNX11. SNX12 is a SNX3 homologue that also associates with VPS35 (Fig. 1b); SNX11 is a closely related SNX-PX protein, having the highest degree of sequence similarity with SNX3/SNX12, but lacks the ability to bind to VPS35 (Fig. 1b). From this we isolated several site-directed mutants in SNX3 targeting individual amino acids or clusters of amino acids (Fig. 1b). When expressed as amino-terminal tagged GFP fusion proteins, all the GFP-SNX3 mutants retained their localisation to VPS35-positive endosomes (Fig. 1c and Supplementary Fig. 1). This is consistent with these mutants not causing perturbed PX domain binding to phosphatidylinositol 3-monophosphate, or a major misfolding of the resultant modified SNX3 protein. Immunoprecipitation of each GFP-SNX3 mutant from HEK293T cells, using the highly efficient GFP-nanotrap, established that substitution of a double arginine motif, GFP-SNX3(p.RR-AA), and deletion of residues 22 to 28 immediately preceding the first β1 strand, SNX3(p.Δ22-28), both led to loss of VPS35 binding (Fig. 1d). Within this unstructured region, a single mutation of the highly conserved tyrosine at position 22, GFP-SNX3(p.Y22A), also led to a complete loss of VPS35 binding (Fig. 1d).

Switching of the surface exposed^30^ EID^32^ sequence on the β1 strand of SNX3 for the corresponding^18^ TIR^20^ residues in SNX11, GFP-SNX3(p.EID-TIR), and the switch of glutamic acid at position 50 on the β2 strand of SNX3 for the corresponding lysine in SNX11, GFP-SNX3(p.E50K), also abrogated VPS35 binding (Fig. 1d). None of the remaining mutations within the PX domain itself led to a complete loss of VPS35 binding (Fig. 1d). Mapping of these residues onto the NMR structure of full length SNX3^34^, revealed that those residues involved in VPS35 binding lay along an exposed surface, composed of the unstructured amino-terminal region and the β1 and β2 strands at the beginning of the PX domain (Fig. 1e). We noted that the unstructured carboxy-terminal tail is also predicted to lay in close proximity to this binding surface. We therefore generated three more constructs, containing mutations within the unstructured carboxy-terminal tail. Only one, mutating an arginine at position 160 to an alanine, GFP-SNX3(p.R160A), led to a loss of VPS35 binding (Fig. 1d). Together, these data establish that conserved residues within the unstructured amino-terminal region of human SNX3 and the β1 and β2 strands are the primary determinants for binding to VPS35, while the extreme carboxy terminus may also influence binding efficiency. These data confirm and extend the recent findings into the mechanism of SNX3′s interaction with VPS35^30,32^.

In *C. elegans*, SNX3-retromer mediates endosome-to-TGN retrieval of the Wls orthologue MIG-14^14,15^. In mutants of *Ce-snx-3* and *Ce-vps-35*, MIG-14 fails to be retrieved from the endosomal system and is degraded in lysosomes^14,15^. Consequently, steady state levels of MIG-14 are reduced, leading to defects in Wnt secretion and a range of Wnt related phenotypes^14,15,35,36^. Among these phenotypes is a defect in the migration of the QL neuroblast descendants (QL.d). During the first stage of larval development, signalling through the Wnt ligand EGL-20 induces the QL.d to migrate towards positions in the posterior part of the animal^37,38^. In the absence of EGL-20, or in mutants that interfere with Wnt secretion, the QL.d migrate in the opposite, anterior, direction^9,13,14,37^ (Fig. 1f). To translate our in vitro findings of the mechanism of SNX3 binding to VPS35 into the in vivo context of SNX3-retromer mediated endosomal sorting of WIs, we utilised CRISPR/Cas9 gene editing to introduce the loss-of-function SNX3(p.Y22A) mutation into the endogenous *Ce-snx-3* locus and examined the effect on QL.d migration. Null mutants of *Ce-snx-3* show a fully penetrant defect in QL.d migration^14^. Similarly, we found that in 81 ± 4.7% (s.d.) of *Ce-snx-3(hu256[Y22A])* mutants (*n* = 202), the QL.d localised anteriorly, consistent with an essential function of this conserved tyrosine position in EGL-20/Wnt signalling (Fig. 1g). Together, these data establish the essential importance of the direct binding of SNX3 to VPS35 for the in vivo SNX3-retromer mediated endosomal sorting of Wls.

### Proteomic quantification of the SNX3 interactome

**Fig. 2:**
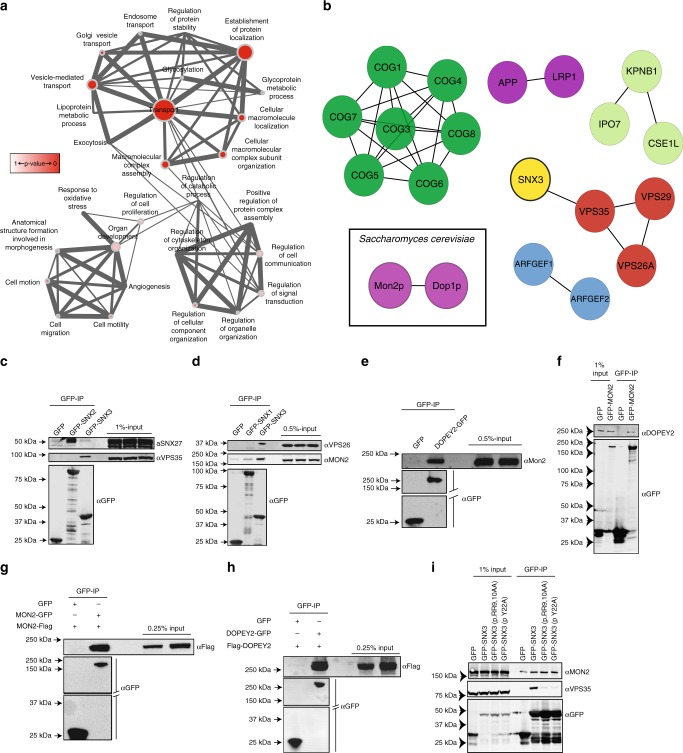
SNX3 engages a flippase complex proposed to regulate membrane deformation. **a** Functional annotation analysis of proteins identified in the SNX3 SILAC proteomics using Gene Ontology annotations with a greater than 3-fold enrichment and with a minimum of two peptides revealed a preponderance of proteins involved in “transport” (47/106), “vesicle mediated transport” (33/106) and “establishment of protein localisation” (33/106). The larger the node the greater the number of proteins classified in that category, while the thicker the edge between nodes the greater the overlap of proteins within those classifications. **b** Network analysis of putative SNX3 interactome components classified within the “transport” node using the STRING database. **c** SNX3 does not engage SNX27. HEK293T cells transiently expressing GFP-SNX2 and GFP-SNX3 were immuno-precipitated and analysed for binding to endogenous SNX27. **d** SNX3 but not SNX1 associates with MON2. Cell extracts derived from RPE1 cells lentivirally transduced with GFP, GFP–SNX3 or GFP–SNX1, were subjected to a GFP nanotrap and subsequently analysed for binding to MON2. **e**, **f** DOPEY2 associates with MON2. Cell extracts derived from HEK293T cells transiently transfected with GFP, DOPEY2-GFP or GFP-MON2 were subjected to a GFP nanotrap and subsequently blotted with antibodies raised against endogenous MON2 and DOPEY2. **g** MON2 can self-associate. Cell extracts derived from HEK293T cells transiently transfected with GFP and MON2-FLAG or GFP–MON2 and MON2-FLAG were subjected to a GFP nanotrap and subsequently blotted with a FLAG antibody. **h** DOPEY2 can self-associate. Cell extracts derived from HEK293T cells transiently transfected with GFP and DOPEY2-FLAG or DOPEY2-GFP and DOPEY2-FLAG were subjected to a GFP nanotrap and subsequently blotted with an anti-FLAG antibody. **i** Binding of SNX3 to endogenous MON2 occurs independently of SNX3′s ability to bind to retromer. Cell extracts derived from HEK293T cells transiently transfected with GFP or GFP-tagged wild-type or mutant forms of SNX3 were subjected to a GFP nanotrap and subsequently blotted with an antibody raised against endogenous MON2. C-I are representative blots from three independent biological replicates

To delve further into the mechanism of SNX3-retromer mediated sorting of Wls, we turned to SILAC (stable isotope labelling with amino acids in cell culture)-based proteomics to identify and quantify the interactome of human SNX3. To ensure a low level of transgene expression, we stably transduced retinal pigment epithelial (RPE-1) cells with either GFP or GFP-SNX3, and grew them in medium containing unlabelled (R0K0) or medium-labelled (R6K4) amino acids for approximately six doublings, prior to highly efficient GFP-trap immuno-isolation^39^. The co-immuno-precipitating proteins were subsequently identified through LC-MS/MS spectrometry^40–43^. From 1,854 quantified proteins (Supplementary Data 1), data was filtered based on two criteria: a > 3-fold enrichment in the GFP-SNX3 over GFP interactome; and protein quantification achieved from two or more peptides (Supplementary Data 2). Gene ontology analysis of the resulting 106 proteins revealed that the majority of these interactors had a role in ‘transport’ (Fig. 2a). Network interaction analysis confirmed previous work^14,15,30–32^, showing the association of SNX3 with the retromer components VPS26A, VPS29 and VPS35 (Fig. 2b, Table 1, and Supplementary Data 2). Importantly, there was no enrichment of the retromer SNX-BARs (SNX1, SNX2, SNX5, SNX6 or SNX32), which is consistent with the distinct functional roles of the SNX3-retromer and SNX-BAR-retromer^14,15^. Extending this, the SNX3 interactome was not enriched in SNX27, the core component of the SNX27-retromer^41,44,45^, data that we confirmed by western analysis (Fig. 2c). Consistent with this, genetic manipulation of *Ce-snx-27* did not affect Wnt signalling in *C. elegans* (Supplementary Fig. 2A). Overall, data from the SNX3 interactome are entirely consistent with the in vitro and in vivo molecular and functional distinction between the SNX3-retromer, and the SNX-BAR-retromer and SNX27-retromer.

**Table 1:** Fold enrichment and number of peptides detected for components of the retromer and MON2 and DOPEY2 in the SNX3 proteomics

# Peptides	SNX3 versus GFP	SNX3 Interactor
	Heavy/light	Protein
17	9.159	VPS35
5	6.980	VPS29
5	100.000	VPS26A
12	5.355	MON2
2	100.000	DOPEY2

### SNX3 interacts with a conserved MON2-DOPEY2 complex

In the context of how SNX3-retromer may couple Wls enrichment to membrane re-modelling during vesicular carrier formation^14^, the SNX3 interactome lacked proteins with predicted membrane re-modelling domains (e.g., BAR domains, ENTH domains) (Supplementary Data 2). Network analysis did however, highlight the enrichment of MON2 and DOPEY2 (Fig. 2b, Table 1, and Supplementary Data 2). Their *S. cerevisiae* equivalents, Mon2p and Dop1p, interact genetically and physically^46–48^, and together, couple to the endosome and late Golgi resident integral membrane protein Neo1p^49,50^, to form an endosomal membrane remodelling complex^48^. Neo1p is considered a P4-type aminophospholipid translocase (flippase)^50,51^. Members of this evolutionary conserved family drive the ATP-dependent transport of phospholipids from the extracellular, or luminal, leaflet to the cytosolic leaflet of biomembranes;^52^ phosphatidylethanolamine may be the preferred substrate of neo1p^53,54^. The net result of directed phospholipid flipping is two-fold. First, the lateral expansion of one leaflet relative to the other, which, if spatially restricted, leads to lipid asymmetry that supports membrane re-modelling in the form of positive membrane curvature. Secondly, the resultant alteration in phospholipid composition is sensed by cytosolic accessory factors, leading to their recruitment to the cytosolic facing leaflet^52,55^. Mon2p and Dop1p are proposed to function as accessory proteins for Neo1p during membrane remodelling and for the formation of vesicular transport carriers^48,56^. Given such a precedent, and the reported genetic link between *Ce-tat-5* and *Ce-mon-2* (the *C. elegans* orthologues of neo1p and mon2p) in retromer-dependent transport in the control of cortical asymmetry of β-catenin during asymmetric division^57^, we postulated that by engaging the Neo1p-Mon2p-Dop1p complex, SNX3-retromer may support the formation of the observed vesicular Wls-enriched transport carriers^14^.

To validate the proteomic data, the interaction between GFP-SNX3 and endogenous MON2 was confirmed by co-immunoprecipitation; this was observed to be specific for SNX3 over the SNX-BAR-retromer component SNX1 (Fig. 2d). This is consistent with the functional distinction between the SNX-BAR-retromer and the SNX3-retromer. Further studies will be required to establish whether the interaction is direct. We then sought to determine to what extent the biochemical features of Mon2p and Dop1p are conserved in their mammalian counterparts; we did this through overexpression and immuno-precipitation experiments. Consistent with the formation of a Mon2p-Dop1p complex in yeast^46^, immuno-precipitation of GFP-DOPEY2 resulted in the co-immunoprecipitation of endogenous MON2 (Fig. 2e). Conversely, immunoprecipitation of GFP-MON2 co-immunoprecipitated endogenous DOPEY2 (Fig. 2f). Furthermore, and again entirely consistent with their yeast counterparts^46–48,56^, immunoprecipitation of GFP- and FLAG-tagged MON2 and DOPEY2 revealed the ability of these proteins to self-associate (Fig. 2g, h). Therefore, at least between yeast and humans, the ability to form higher-ordered oligomeric assemblies is evolutionary conserved; this may aid membrane remodelling during the formation of transport carriers^48^. Finally, the two SNX3 mutants that lack the ability to bind to VPS35, GFP-SNX3(p.Y22A) and GFP-SNX3(p.RR-AA), both retained their ability to associate with MON2, revealing the retromer-independent nature of this interaction (Fig. 2i).

### In vitro analysis of SNX3-retromer mediated sorting of Wls

**Fig. 3:**
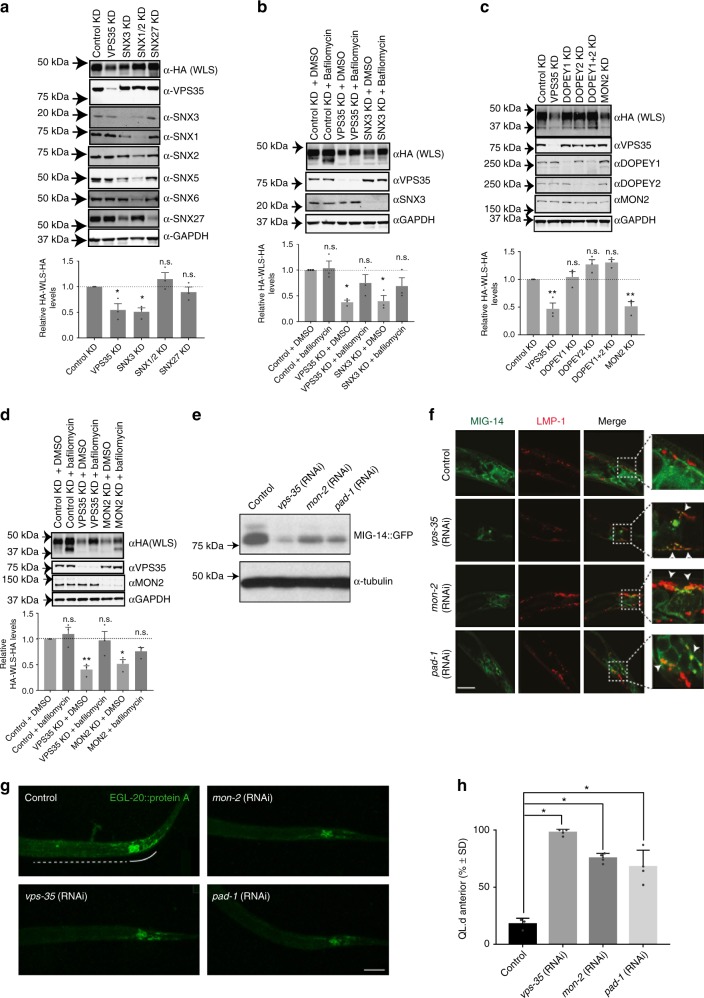
MON2-DOPEY2 complex is required for SNX3-retromer-mediated sorting of Wls. **a** RNAi-mediated suppression of VPS35, SNX3, SNX1 and SNX2, or SNX27 in RPE-1 cells stably expressing HA-WLS and quantification of HA-WLS levels. **b** Inhibition of lysosomal degradation through Bafilomycin A1 treatment (100 nM for 16 h) restored total protein levels of HA-WLS following RNAi-mediated suppression of VPS35 or SNX3. **c** RNAi-mediated suppression of VPS35, DOPEY1, DOPEY2, DOPEY1 + DOPEY2 or MON2 reveals that the total protein levels of HA-WLS is dependent on the function of VPS35 and MON2. **d** Bafilomycin A1 treatment (100 nM for 16 h), inhibiting lysosomal degradation, prevents loss of HA-WLS protein levels following VPS35 and MON2 suppression using RNAi. **a**–**d** Data presented as mean +/− s.e.m. from three independent biological replicates. Significance was determined using a one-way ANOVA with a post-hoc Dunnett’s test; *p < 0.05. **e** Representative western blot of endogenously expressed MIG-14::GFP (*huSi2*) protein levels in animals treated with control, *vps-35*, *mon-2* or *pad-1* RNAi. **f** Confocal imaging of MIG-14::GFP (green, *huIs72*) and the late endosomal and lysosomal marker LMP-1::mCherry (red, huEx149) in L1 larvae. The tail area, which includes the EGL-20 producing cells, is shown. Arrows indicate regions of colocalization. In all images, anterior is left and dorsal is up. Scale bar is 10 µm. **g** Staining of EGL-20::protA with rabbit-anti-goat-Alexa647 in L1 larvae. The EGL-20 producing cells are indicated with a solid line, the punctate gradient that is formed by EGL-20::protA is indicated with a dashed line. In all images, anterior is left and dorsal is up. Scale bar is 10 µm. **h** Systemic knock down of *mon-2* or *pad-1* interferes with the EGL-20/Wnt dependent posterior migration of the QL descendants (QL.d) in a *vps-29(tm1320)* sensitised genetic background. The percentage of animals with anteriorly displaced QL.d is shown (data are presented as mean +/− SD and include results from four independent biological replicates, with *n* ≥ 25 per replicate) *p = 1.158 × 10^–6^ for *vps-35* RNAi, *p = 1.44 × 10^–5^ for *mon-2* RNAi and *p = 0.0024 for *pad-1* RNAi (Student’s *t*-test)

To establish an in vitro cell culture-based assay for Wls trafficking, we took advantage of the development of a Wls expression construct. This construct was engineered to include an amino-terminal HA tag in the first extracellular loop, and a carboxy-terminal HA tag that occludes the ER retention motif, thereby optimising trafficking of Wls through the endosomal network (herein termed HA-Wls)^31^ (Supplementary Fig. 3A). When lentivirally transduced into RPE-1 cells, HA-Wls displayed a steady-state subcellular localisation defined by enrichment at TGN46-labelled *trans*-Golgi network (TGN) and VPS35 decorated endosomes (Supplementary Fig. 3B and 3C). Targeted RNAi-mediated suppression, coupled with quantitative western analysis, revealed a significant reduction in the whole-cell steady-state levels of HA-Wls upon suppression of either SNX3 or VPS35 (Fig. 3a). This decrease in the whole-cell level of HA-Wls upon SNX3 or VPS35 suppression was reverted by incubation with bafilomycin to inhibit lysosomal-mediated degradation, consistent with the mis-sorting of internalised Wls into the degradative pathway (Fig. 3b). A similar decrease in Wls levels was not observed upon dual suppression of SNX1 and SNX2 (components of the SNX-BAR-retromer)^23,24^, or the individual suppression of SNX27 (a component of the SNX27-retromer)^41^, thereby revealing the specific nature of this sorting defect (Fig. 3a). While this assay is limited by the need to overexpress an engineered Wls, the use of lentivirally transduction to lower HA-Wls expression does constitute a suitable in vitro cell-based assay for following the SNX3-retromer-mediated endosome sorting of Wls and its retrieval away from lysosomal-mediated degradation.

Having established the validity of this assay, we next turned to RNAi-mediated suppression of MON2. This led to a significant reduction in the steady-state level of HA-Wls similar to that observed upon suppression of SNX3 or VPS35 (Fig. 3c). Entirely consistent with a defect in the retrieval of Wls away from lysosomal-mediated degradation, the effect of MON2 suppression on the level of HA-Wls was reverted upon treatment with bafilomycin (Fig. 3d). Interestingly, neither the individual suppression of DOPEY2, a closely related protein DOPEY1, nor the dual suppression of DOPEY1 and DOPEY2, had any detectable effect on the levels of HA-Wls (Fig. 3c). Thus, while MON2 and DOPEY2 can form an evolutionary conserved complex, in human cells the MON2 and SNX3-retromer-dependent endosomal sorting of Wls appears to be functionally independent of either DOPEY protein.

### MIG-14 trafficking requires *C. elegans* MON2 and DOPEY2

To extend our in vitro analysis to a physiological relevant in vivo setting, we knocked down the *C. elegans* MON2 orthologue, *Ce-mon-2*, and the DOPEY2 orthologue, *Ce-pad-1*, using RNA interference (RNAi) and examined whether steady state levels of MIG-14 were changed. Similar to the knock-down of *Ce-vps-35*, depletion of *Ce-mon-2* or *Ce-pad-1* resulted in a significant reduction in total MIG-14 protein levels (Fig. 3e). Furthermore, and again similar to the suppression of *Ce-vps-35*, knock-down of *Ce-mon-2* or *Ce-pad-1* induced clear co-localisation of a functional MIG-14::GFP fusion protein with the late endosomal and lysosomal marker LMP-1::mCherry (Fig. 3f)^14^. Suppression of *Ce-mon-2* or *Ce-pad-1* also led to a reduction in Wnt secretion. The Wnt protein EGL-20 is expressed by a group of cells in the tail and forms a posterior-to-anterior concentration gradient which can be visualised using a functional fusion of EGL-20 with the immunoglobulin domain of protein A (ProtA)^37,14^. In control RNAi treated animals, EGL-20::protA was visible as a punctate staining pattern that ranges from the producing cells in the tail to the mid-body region (Fig. 3g). Knock-down of *Ce-vps-35* resulted in the previously documented clear reduction in EGL-20::protA staining^9^. Knock-down of *Ce-mon-2* or *Ce-pad-1* resulted in a similar reduction in EGL-20::protA staining, indicating that *Ce-mon-2* and *Ce-pad-1* are, like the SNX3-retromer, required for Wnt secretion in vivo.

Next, we examined the effect of this reduction in EGL-20 secretion on QL.d migration. Because QL.d migration is relatively insensitive to changes in EGL-20 levels^14^, we used a sensitised genetic background (a mutation in the retromer subunit gene *vps-29)* which partially reduces MIG-14 retrieval and enhances the QL.d migration phenotype of Wnt secretion pathway components^14,58^. Similar to knock-down of *Ce-vps-35*, RNAi of *Ce-mon-2* or *Ce-pad-1* induced a significant increase in the percentage of animals with anteriorly displaced QL.d (Fig. 3h). While the human DOPEY2 appears not to be functionally required for MON2-dependent sorting of HA-Wls, the MON2-PAD1 complex in *C. elegans* is required for in vivo Wnt morphogenic gradient formation. Together, these data provide evidence of a genetic linkage between SNX3-retromer and the MON-2-PAD-1 complex in this process.

### MON2-DOPEY2 associates with the putative flippase ATP9A

**Fig. 4:**
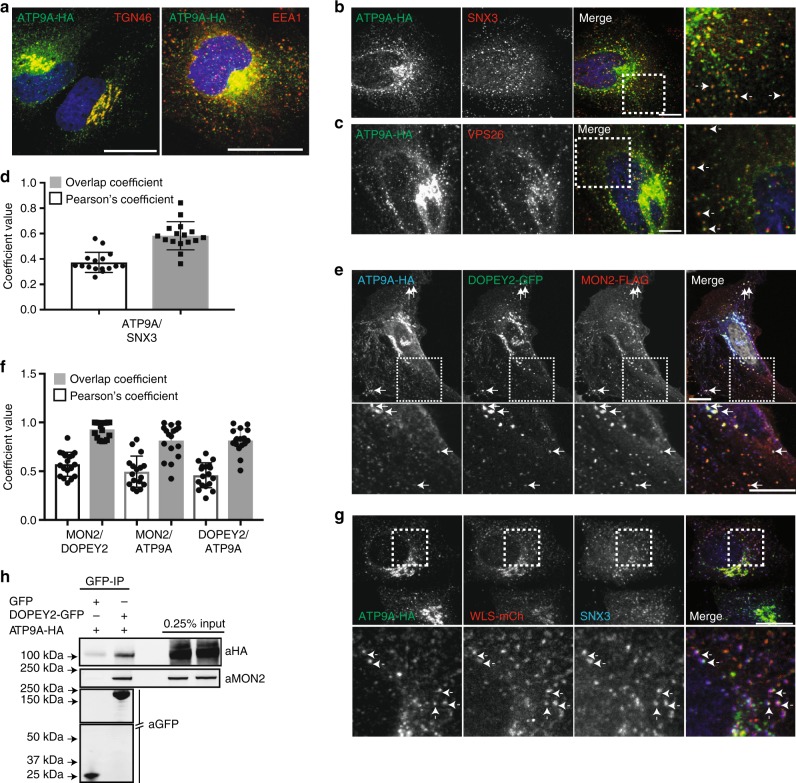
ATP9A co-localises with MON2 and DOPEY2 on SNX3-retromer endosomes. **a** ATP9A-HA is found on early endosomes and at the TGN. HeLa cells were transiently transfected with ATP9A-HA and fixed and stained with an anti-HA (green) antibody and antibodies detecting either endogenous TGN46 or EEA1 (red). The blue staining indicates DAPI. Scale bar represents 10 μm. **b**, **c** ATP9A-HA colocalises on endosomes labelled for the SNX3-retromer components SNX3 and VPS26. HeLa cells were transiently transfected with ATP9A and subsequently fixed and stained for VPS26 and SNX3. Arrows indicate endosomes co-labelled. The blue staining indicates DAPI. Scale bar represents 10 μm. **d** Quantification of the co-localisation between ATP9A and SNX3 (Pearsons 0.37 ± 0.08, Overlap Coefficient 0.58 ± 0.11, *n* = 15 technical replicates). Error bars indicate s.e.m. **e** HeLa cells were transiently transfected with ATP9A-HA, DOPEY2-GFP and MON2-FLAG and subsequently fixed and stained with anti-HA and anti-FLAG antibodies. Arrows indicate endosomes labelled for all three components. Scale bar represents 10 μm. **f** Quantification of the co-localisation between MON2 and DOPEY2 (Pearsons 0.57 ± 0.12, Overlap Coefficient 0.92 ± 0.08, *n* = 18 technical replicates), MON2 and ATP9A (Pearsons 0.49 ± 0.15, Overlap Coefficient 0.81 ± 0.17, *n* = 17 technical replicates) and DOPEY2 and ATP9A (Pearsons 0.45 ± 0.12, Overlap Coefficient 0.81 ± 0.11, *n* = 18). Error bars indicate s.e.m. **g** HeLa cells were transiently transfected with ATP9A and WLS-mCherry and subsequently fixed and stained for endogenous SNX3 and HA. Arrows indicate endosomes labelled for all three components. Scale bar represents 10 μm. **h** Representative blot showing DOPEY2-GFP binds both MON2 and ATP9A-HA. Cell extracts derived from HEK293 cells transiently transfected with GFP and ATP9A-HA or DOPEY2-GFP and ATP9A-HA were subjected to a GFP nanotrap and subsequently blotted with antibodies raised against HA and MON2

The association of the Mon2p-Dop1p complex with the putative flippase Neo1p has only been described in yeast^46,48^. Neo1p has two human orthologues, ATP9A and ATP9B. ATP9B-HA, when expressed in HeLa cells, is localised exclusively to the Golgi apparatus^59,60^. In contrast, ATP9A-HA was observed at the TGN and on endosomes, the latter being consistent with its described role in endosomal recycling^60^ (Fig. 4a). ATP9A-HA showed extensive co-localisation with endosome-associated SNX3 and the retromer component, VPS26 (Fig. 4b–d). Co-expression of MON2-Flag and DOPEY2-GFP with ATP9A-HA revealed the co-localisation of proteins to ATP9A-labelled endosomes (Fig. 4e, f). In addition, ATP9A was observed on endosomes positive for both SNX3 and the cargo, Wls (Fig. 4g). Consistent with their endosomal co-localisation and building on evidence in yeast of a Neo1p-Mon2p-Dop1p complex, immunoprecipitation of DOPEY2-GFP resulted in the co-precipitation of ATP9A-HA and endogenous MON2 (Fig. 4h). Overall, ATP9A is associated with MON2 and DOPEY2 on SNX3-retromer decorated endosomes, a location entirely consistent with the functional requirement of MON2 and ATP9A (see below) in the SNX3-retromer mediated endosomal sorting of Wls.

### ATPase cycle of ATP9A required for Wnt gradient formation

**Fig. 5:**
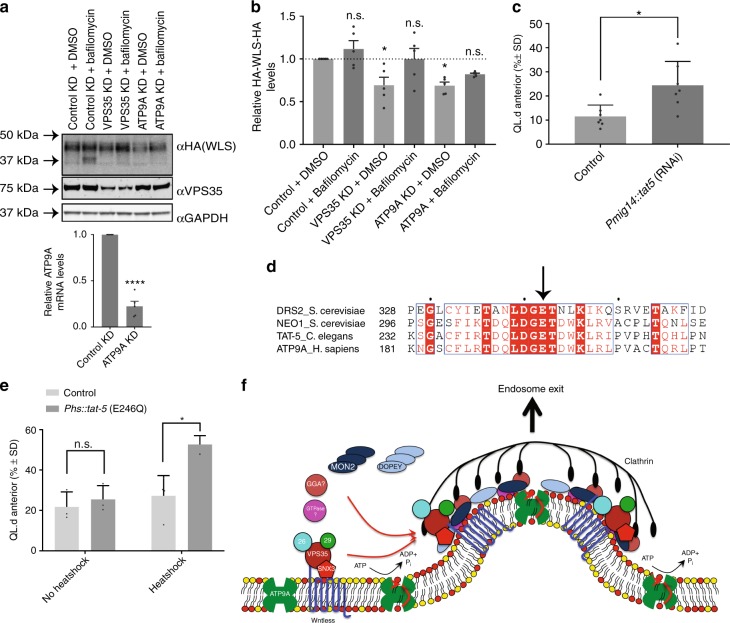
ATP9A is required for Wls sorting and is required for Wnt gradient formation. **a** RPE-1 cells, stably expressing HA-WLS, under RNAi-mediated suppression of VPS35 or ATP9A were treated with Bafilomycin A1 (100 nM for 16 h). Knock-down of ATP9A was confirmed through RT-qPCR. **b** Quantification of HA-WLS protein levels reveal a similar loss of HA-WLS through VPS35 or ATP9A suppression that is reverted upon blocking of lysosomal-mediated degradation using Bafilomycin A1. Data presented as mean +/− s.e.m. from five independent biological replicates. Significance was determined using a one-way ANOVA followed by a post-hoc Dunnett’s test; *p < 0.05. **c** Tissue specific RNAi of *tat-5* in Wnt producing cells (*Pmig-14::tat-5* RNAi) in a *vps-29(tm1320)* sensitised mutant background. The percentage of animals with anteriorly displaced QL.d is shown (data are presented as mean +/− SD and include results from seven independent biological replicates, *n* ≥ 24 per replicate) *p = 0.013 (Student’s *t*-test). **d** Sequence alignment of Drs2p with Neo1p homologues highlighting the conserved catalytic glutamic acid residue essential for ATPase activity. **e** Overexpression of catalytically inactive TAT-5(E246Q) in a *vps-29(tm1320)*sensitised mutant background. The percentage of animals with anteriorly displaced QL.d is shown (data are presented as mean +/− SD and include results from three independent biological replicates, *n* ≥ 30 per replicate) *p = 0.0089 (Student’s *t*-test). **f** Schematic representation of our working model: SNX3-retromer, coupled to MON2/DOPEY2, enriches Wntless into nascent carriers, formed through the flippase activity of ATP9A

To reveal the functional importance of ATP9A for the endosomal sorting of Wntless, we first utilised the in vitro HA-Wls assay. The RNAi-mediated suppression of ATP9A led to a decrease in the steady-state levels of HA-Wls, a decrease that was reverted upon treatment with bafilomycin (Fig. 5a, b). ATP9A is therefore required for the SNX3-retromer mediated retrieval of Wls away from lysosomal-mediated degradation. To extend this analysis, we turned to genetic analysis in *C. elegans*. Systemic RNAi of the ATP9A orthologue, *Ce-tat-5*, is early embryonic lethal^61^. To examine whether *Ce-tat-5* is also required for EGL-20 signalling, we circumvented the essential function of *Ce-tat-5* during embryonic development by specifically knocking down *Ce-tat-5* in Wnt producing cells, using transgene-mediated RNAi. Knock-down of *Ce-tat-5* in a *vps-29(tm1320)* sensitised mutant background resulted in a significant defect in QL.d migration (Fig. 5c), similar to *Ce-*vps-35 knock-down (Supplementary Fig. 2B-C). This supports the notion that TAT5 functions together with MON2 and PAD1 in SNX3-retromer-mediated Wls retrieval and Wnt secretion.

Neither ATP9A, TAT5 or Neo1p have biochemically been shown to possess flippase activity. However, strong genetic evidence in *C. elegans* is consistent with TAT5 regulating the asymmetry of phosphatidylethanolamine^61^ and, in budding yeast, inactivation of Neo1p leads to preferential exposure of phosphatidylethanolamine on the outer leaflet^53^, again consistent with a role in phospholipid translocation. In the related yeast flippase Drs2p, a Drs2p(E342Q) mutation blocks the ATPase cycle at the *E*_2_P conformation, and hence inhibits phospholipid translocation across the bilayer^62,63^. To examine the importance of the ATPase cycle, and hence putative flippase activity of TAT5 in Wnt gradient formation, we generated the corresponding mutant (Fig. 5d). Overexpression of the TAT5(E246Q) mutant in a *vps-29(tm1320)* sensitised mutant background interfered with QL.d migration (Fig. 5e). The TAT5 ATPase cycle, and hence its putative flippase activity, is therefore required for Wnt signalling. Taken together, these results are consistent with a requirement of the MON2-PAD-1 complex and the putative flippase activity of TAT5 in the in vivo SNX3-retromer-mediated Wls retrieval and Wnt secretion in *C. elegans*.

## Discussion

By defining the mechanism of SNX3 binding to the retromer VPS35 subunit, we have established the essential role of this interaction for the in vivo retrieval of Wls and the maintenance of Wnt secretion in *C. elegans*. Through utilising unbiased and quantitative proteomic procedures, we have defined the human SNX3 interactome and in so doing have identified a number of potentially important functional interactions. In the present study, we have focused on the question of how the SNX3-retromer couples to an evolutionary conserved membrane remodelling complex to elicit the formation of vesicular transport carriers enriched in the cargo protein Wls^14^. From a bioinformatic analysis of the quantified SNX3 interactome, we identified MON2 and DOPEY2, whose orthologues in yeast are known components of an endosomal membrane remodelling complex that contains the putative phospholipid flippase neo1p^48^. We have shown that MON2 and DOPEY2 associate with ATP9A (an orthologue of neo1p), to form an analogous complex in mammalian cells; SNX3 associates with this complex in a manner that is distinct to the mechanism of SNX3 binding to retromer. The development of a human cell culture system for studying the endosomal sorting of Wls has established a functional link with MON2 and ATP9A in this pathway; the precise role and importance of DOPEY2 remains unclear. In extending our analysis into *C. elegans*, we have established the in vivo importance of the corresponding MON2-PAD1-TAT5 complex in SNX3-retromer-mediated Wls sorting and Wnt signalling in this model organism. Importantly, while attempts at reconstituting the in vitro phospholipid translocation activity of neo1p have so far not been successful because of technical reasons^53^, we have shown that the in vivo ATPase activity of TAT5 is required for the MON2-PAD1-TAT5 dependent SNX3-retromer mediated sorting of Wls. When taken with the published role that the disruption of neo1p, TAT5 and ATP9A all lead to phenotypes consistent with their role in the flipping of phospholipids^53,54,60,61,64,65^, our data argue that the phospholipid translocation activity of these P4-ATPases is an important component in the endosomal sorting of Wls. Furthermore, in yeast, Snx3p recruits Neo1p to mediate recycling of the known yeast Snx3p-cargo A-ALP, fully supports a role for ATP9A in human SNX3-retromer mediated sorting^65^.

Building on evidence from yeast^46–48,50,56,65^, we therefore propose a working model (Fig. 5f) in which TAT5/ATP9A and the MON2-PAD1 complex are associated with SNX3-retromer on the endosomal network. The role of DOPEY proteins in the corresponding mammalian MON2-DOPEY2 complex remains to be clarified. However, recently published data examining the role of these proteins in *C. elegans* indicate that PAD1 and MON2 may have separate functions, with TAT5/PAD1 maintaining PE asymmetry at the cell surface, and TAT5-MON2 regulating endosomal trafficking^66^. Here, the ATPase cycle and putative flippase activity of ATP9A/TAT5 generates an asymmetric membrane structure that, in expanding the surface area of the cytosolic leaflet over the luminal leaflet, within a diffusion restricted sub-domain, induces initial membrane bending into the cytosol. In addition, the concomitant alteration in phospholipid composition of the cytosolic leaflet that arises from the flipping of specific lipids (*e.g*. phosphatidylethanolamine), provides an environment for the membrane recruitment and assembly of coat proteins and other accessory factors, including the MON2-PAD1 complex, that aid formation of vesicular transport carriers. These events are co-ordinated with capture and enrichment of the Wls cargo through the SNX3-retromer, as recently structurally defined for SNX3-retromer binding to the divalent cation transporter, DMT1-II^32^. Additional accessory proteins are also likely to assist in the formation of the transport carrier. Indeed, both yeast and human Mon2p/MON2 associate with GGA (Golgi-localising, γ-adaptin ear domain homology, ARF-binding protein) clathrin adaptors^56^, an interaction that is entirely consistent with the observation that Wls-containing SNX3-labelled vesicular transport carriers are clathrin-decorated^14^. Testing aspects of this model and how it has functionally evolved from yeast to humans will provide further mechanistic details of SNX3-retromer mediated sorting and elucidate the role of the MON2-PAD1 complex as an evolutionary conserved endosomal coat, and, more broadly, the role of flippases in carrier formation during membrane transport.

In *C. elegans*, the retromer-dependent sorting of the bone morphogenetic protein (BMP) type I receptor, SMA-6, is also severely perturbed in mutants of *Ce-snx-3* but not *snx-1* or *Ce-snx-27*^67^. Our data suggests that the SNX3-retromer mediated endosomal sorting of SMA-6 may be dependent on Ce-*mon-2*, Ce-*pad-1* and Ce-*tat-5*, and further experiments will be required to directly test this. Whether this functional link can be extended to the SNX3 and retromer-dependent recycling of the transferrin receptor during transport of iron in vertebrate erythropoiesis will also require further study^68^. Recent published evidence that ATP9A is required for transferrin receptor recycling in HeLa cells is entirely consistent with such an involvement^60^. Interestingly, this study also revealed that depletion of ATP9A perturbed the endosomal recycling of the SNX27-retromer cargo protein GLUT1, suggesting that in this experimental system the role of ATP9A may also be extended to SNX27-dependent cargo sorting^41^.

Our unbiased proteomic analysis has also identified a number of additional SNX3 interactors that may provide further insight into SNX3′s function in endosomal biology. These include, but are not restricted to, the conserved oligomeric Golgi (COG) complex, a Golgi and TGN-localised multi-subunit tethering complex that has established roles in intra-Golgi transport and the capture and organisation of endosome-derived transport carriers for fusion at the TGN^69,70^. Equally, the enrichment of VAC14 and FIG4, two components of a heterotrimeric complex that, by regulating the interconversion of phosphatidylinositol 3-monophosphate (PtdIns(3)P) and phosphatidylinositol 3,5-bisphosphate (PtdIns(3,5)P_2_), controls early-to-late endosomal maturation^71^ and syndecan-1 and syntenin, proteins that together regulate intraluminal budding of endosomal membranes^72^, may provide mechanistic insight into the proposed role of SNX3 in endosomal maturation^73^ and intraluminal vesicle formation^74^. Further work will be required to validate these interactions and provide functional insight into their potential significance.

In summary, our data provides molecular insight into how the SNX3-retromer orchestrates cargo capture and enrichment with membrane remodelling during endosomal sorting of Wls^14,15^, and adds to our understanding of the molecular diversity between SNX-BAR and SNX3-retromers^17^, paving the way for further detailed mechanistic analysis of this important cargo sorting pathway.

## Methods

### Cell culture

HeLa, HEK293T and RPE1 cells were maintained in DMEM (Gibco-Invitrogen) plus 10% (v/v) foetal calf serum (Sigma-Aldrich) and penicillin/streptomycin (PAA). The cell lines were originally sourced from ATCC and regularly tested for mycoplasma contamination.

### Immunofluorescence analysis

Plasmids containing the stated constructs were transfected using Lipofectamine LTX reagent (Invitrogen). 48 h after transfection cells were fixed in 0.1 M phosphate buffer containing 4% (w/v) paraformaldehyde for 10 min on ice and permeabilized with 0.1% (v/v) Triton X-100 for 5 min. Thereafter, cells were incubated with 0.5% (w/v) BSA for 30 min followed by incubation with the indicated primary antibodies and subsequent incubation with secondary antibodies (Molecular Probes). For nuclear staining, DAPI was used. Images were recorded on a Leica SPE or a Leica AOBS-SP2 confocal microscope.

### Immunoprecipitation and western blotting analysis

HEK293T cells were grown to 85% confluence in 15 cm dishes prior to transfection with 10 µg of plasmid DNA using PEI (Sigma-Aldrich), and incubated for 48 h prior to immunoprecipitation using GFP nanotrap beads (Chromotek)^40–43^. Western blots were performed using standard procedures. Detection was carried out on a Licor Odyssey Infrared scanning system using fluorescently labelled secondary antibodies. Uncropped versions of the blots are shown in Supplementary Fig. 4.

### SILAC interactome analysis

All SILAC reagents were sourced from Thermo Fisher; except for dialysed FBS (Sigma). Lentivirally transduced GFP and GFP-SNX3 RPE1 cells were grown in SILAC DMEM for at least 6 passages to achieve full labelling. GFP-expressing control cells were grown in unlabelled medium containing regular arginine and lysine (R0, K0). GFP-SNX3-expressing cells were grown in medium containing ^13^C_6_-arginine and 4,4,5,5-D4-lysine (R6, K4). GFP was precipitated with GFP-trap beads (Chromotek) for 60 min. The beads were pooled, proteins were eluted in sample buffer, separated on Nupage 4–12% precast gels (Invitrogen) and subjected to LC–MS/MS analysis on an Orbitrap Velos (Thermo) mass spectrometer^40–43^.

### RT-qPCR

Total RNA was extracted from RPE-1 cells using the RNeasy kit (Qiagen). RT-qPCR analysis was undertaken using a SuperScript III Platinum SYBR Green One-Step qRT-PCR kit (Invitrogen). ATP9A fragments were amplified using the following primers: ATP9A forward, 5′-gcctcaccaagatcctctttgg-3′; ATP9A reverse, 5′-ggttcacgcgcaaactaatggg-3′. β-actin was used to normalise the total mRNA in each sample.

### RNAi- mediated suppression

Lentivirally transduced RPE-1 cells stably expressing HA-WLS^31^ were transfected with the following siRNA sequences: ON-TARGET plus nontargeting control pool (GE Healthcare; sequences: 5′-UGGUUUACAUGUCGACUAA-3′, 5′-UGGUUUACAUGUUGUGUGA-3′, 5′-UGGUUUACAUGUUUUCUGA-3′, and 5′-UGGUUUACAUGUUUUCCUA-3′); SNX27: ON-TARGET plus human SNX27 siRNA pool; (GE Healthcare; sequences: 5′-CAAAUUAGCUGCACGUAUA-3′, 5′- GAGUAUAGAUUCCGGCUCA-3′, 5′-GGAAUAUGGUUGAUUAGAU-3′, 5′-GAGUACAAAACGUGAGAAU-3′); MON2: 5′-CAUGCAGAUAAUGUAUCCAGCUATT-3′; SNX3: 5′-AACAAGGGCUGGAGCAGUYYATT-3′; DOPEY2: 5′-UUGGCAAACUCAACAAGGCUCUUTT-3′; VPS35: 5′-GUUGUUAUGUGCUUAGUA-3′ and 5′-AAAUACCACUUGACACUUA-3′; SNX1: 5′-AAGAACAAGACCAAGAGCCAC-3′; SNX2: 5′-AAGUCCAUCAUCUCCAGAACC-3′; ATP9A: 5′-GAAGGUGAAGAGUUCUAACAUTT-3′; DOPEY1: ON-TARGET plus human DOPEY1 siRNA pool (GE Healthcare; sequences: 5′-GGGUAUACAUCAACGAGAA-3′, 5′-GAUGAAAAGGAGCGGGUUA-3′, 5′-CAGCAGUACAAACUU-3′). Cells were reverse transfected using DharmaFECT (GE Healthcare), then transfected again 24 h later. Cells were lysed 72 h after the first transfection.

### Antibodies, constructs and materials

The following antibodies were used in this study: polyclonal rabbit anti-SNX3 (proteintech, 10772-1-AP; 1:100 IF; 1:500 WB), polyclonal rabbit anti-SNX5 (proteintech, 17918-1-AP; 1:1000 WB); monoclonal mouse anti-EEA1 (BD Transduction Laboratories, 610456; 1:200 IF), monoclonal mouse anti-SNX1 (BD Transduction Laboratories, 611482; 1:1000 WB), monoclonal mouse anti-SNX2 (BD Transduction Laboratories, 611308; 1:1000 WB); monoclonal mouse anti-GFP (Roche, mix of clones 7.1 and 13.1; 1:2000 WB); polyclonal rabbit anti-VPS26 (Abcam, Ab137447, 1:1000 WB; Ab23892, 1:200 IF); anti-VPS35 (Abcam, Ab157220; 1:2000 WB; Ab97545, 1:200 IF); monoclonal mouse anti-SNX27 (Abcam Ab77799, 1:500 WB), polyclonal rabbit anti DOPEY1 (Abcam, Ab95458, 1:500 WB); monoclonal mouse anti-HA (Proteintech, 66006-1-1g, 1:1000); monoclonal mouse anti-Flag (Sigma, F3165, 1:1000); polyclonal sheep anti-TGN46 (BioRad, AHP500GT, 1:400 IF) (GeneTex); polyclonal rabbit anti-DOPEY2 (Santa Cruz, Sc-83241, 1:500 WB); monoclonal mouse anti-SNX6 (Santa Cruz, S6324, 1:1000 WB), polyclonal rabbit anti-GAPDH (Sigma, G9545, 1:2000 WB); the rabbit polyclonal anti-Mon2 (1:500 WB) antibody and Mon2-Flag construct were kind gifts from Dr Yoshihiro Kawaoka (University of Wisconsin); fluorescent Alexa secondary antibodies were obtained from Molecular Probes. The construct encoding ATP9A-HA was a gift from Dr. Hye-Won Shin (Kyoto University, Japan). A cDNA clone encoding DOPEY2 was purchased from Fermentas. Both MON2 and DOPEY2 were subcloned into both pEGFP-N1 (Clonetech) and a modified pcDNA3.1 vector which encodes a Flag tag. Site-directed mutagenesis, using Agilent QuikChange Primer Design, was used to introduce the various mutations into pEGFP-C2-SNX3 vector. HA-WLS in a pIRESneo3 vector, a kind gift from Mark von Zastrow (see Varandas et al., 2016^31^ for full cloning details), was subcloned into the XLG lentiviral vector. Bafilomycin A1 was purchased through Sigma (B1793).

### Computational and statistical analysis

Functional gene annotation for the set of SNX3-interacting proteins was performed using the Database for Annotation, Visualisation, and Integrated Discovery (DAVID, version 6.7). The Cytoscape plugin Enrichment Map was used to display overlap between gene ontology terms within Cytoscape (version 2.8.2). Node size was mapped to the number of genes within a GO category and node colour was mapped to the p-value for enrichment of a given category. For network analysis, a network interaction map was built using STRING. The network was visualised in Cytoscape. Minimum replicates were used to gain adequate statistical power. All quantified western blot data are the mean of at least three independent experiments and normalised to GAPDH. Results are shown as a proportion of the control KD or the control KD with DMSO-treatment. Mean and standard error were calculated, followed by one-way analysis of variance (ANOVA) and a posthoc Dunnett’s multiple comparisons test to determine statistical significance using GraphPad Prism 7.00 software. The threshold for significance was *p* < 0.05 and the coding for significance reported is: n.s. *p* > 0.05, *p ≤ 0.05 and **p ≤ 0.01.

### *C. elegans* strains and culture

*C. elegans* strains were cultured at 20 °C using standard conditions as described ^75^. Mutant alleles and transgenes used were: *vps-29(tm1320)*, *muIs32[Pmec-7::gfp]*^76^, *huIs60[Pegl-20::egl-20::protA]*, *huIs72[Pmig-14::mig-14::gfp]*^9^, *huSi2[Pmig-14::mig-14::gfp]*^14^, *huEx149[Pmyo3::lmp-1::mCherry]*^14^, *huEx529[Phs::tat-5(E246Q)]*, *huEx430[Pmig-14::tat-5(RNAi)*], *huEx516[Pmig14::vps-35(RNAi)]* and *huEx518[Pmig-14::gfp(RNAi)]*.

### Generation of *C. elegans snx-3* (Y22A) mutation

The *snx-3* Y22A mutation was made using CRISPR/Cas9 mediated genome editing and a single stranded DNA oligo repair template containing the mutation, as described^77^. Sequences: *snx-3* crRNA: GGACGAGGCATATGCCCCGCgttttagagctatgctgttttg; *snx-3* (Y22A) repair template: CCCTTCAAAACGGCAAACGCTGGACGAGGCAgcTGCCCCGCCGGCCAACTTCCTTGAAATTGAGgtttgaataaaaatcaatgaattagatcaagataacttgcagGTTATCAATCCTATCACTCATGGTGTTGGAAAAATGCGCTACACGGATTACGAAATCAGAATG.

### *C. elegans* RNAi, transgenesis and imaging

Systemic RNAi by feeding and tissue-specific RNAi by transgene mediated expression of double-stranded RNA (dsRNA) was performed as described^78,79^. To express *tat-5*, *vps-35* or *gfp* dsRNA from the *mig-14* promoter, 500 base pair (bp) fragments of coding sequence were PCR amplified from genomic DNA or plasmid template. After PCR fusion to the *mig-14* promoter (in the sense, as well as the antisense orientation), the final PCR products were injected in *vps-29(tm1320); muIs32* animals at a concentration of 7 ng/µl with 7 ng/µl *Pmyo2::mCherry* injection marker and 150 ng/µl pBluescript plasmid DNA, yielding the transgenes *huEx430[Pmig-14::tat-5(RNAi)], huEx516[Pmig14::vps-35(RNAi)]* and *huEx518[Pmig-14::gfp(RNAi)]*.

To generate *Phs::tat-5(E246Q)*, the *tat-5(E246Q)* coding sequence was PCR amplified from pDONR221_TAT-5(E246Q)^61^ using primers (TCCCGGGatgggcaaacggaagaagaacgac and TGCTAGCtcagttgacttt-cgcgtagcttg) that re-introduce a translational start codon at the 5′ end of the *tat-5* coding sequence. After sub-cloning of the resulting PCR fragment into pJET (Promega), *tat-5(E246Q)* was inserted into the heat-shock promoter vector pPD49.78 using XmaI and NheI restriction sites. The construct was injected at 25 ng/µl with 7 ng/µl *Pmyo2::mCherry* injection marker and 150 ng/µl pBluescript plasmid DNA, yielding the transgene *huEx529[Phs::tat-5(E246Q)]*. Synchronised embryos were heat-shocked for 1 h at 33 °C and the QL.d migration phenotype was determined when the animals reached the young adult stage. The final position of the QL descendant PVM was scored relative to the vulva in young adult animals. Antibodies used were anti-goat-Alexa647 (Life Technologies), anti-GFP (BD Livingcolors) and anti-mouse-HRP (GE Healthcare).

## Electronic supplementary material

Supplementary Information

Description of Additional Supplementary Files

Supplementary Data 1

Supplementary Data 2

## Data Availability

The datasets and reagents generated and/or analysed during the current study are available from the corresponding authors on reasonable request. Mass Spectrometry data was deposited at the ProteomeXchange Consortium via the PRIDE partner repository with the dataset identifier PXD010632 (https://www.ebi.ac.uk/pride/archive/projects/PXD010632).
