# Supplementary material for: SNX3-retromer requires an evolutionary conserved MON2:DOPEY2:ATP9A complex to mediate Wntless sorting and Wnt secretion
Source: Nat Commun. 2018 Sep 13;9:3737. doi: 10.1038/s41467-018-06114-3 (PMC6137200; doi:10.1038/s41467-018-06114-3)

SNX3-retromer requires an evolutionary conserved MON2:DOPEY2:ATP9A complex to mediate Wntless sorting and Wnt secretion.

McGough et al., 2018

Supplementary Information

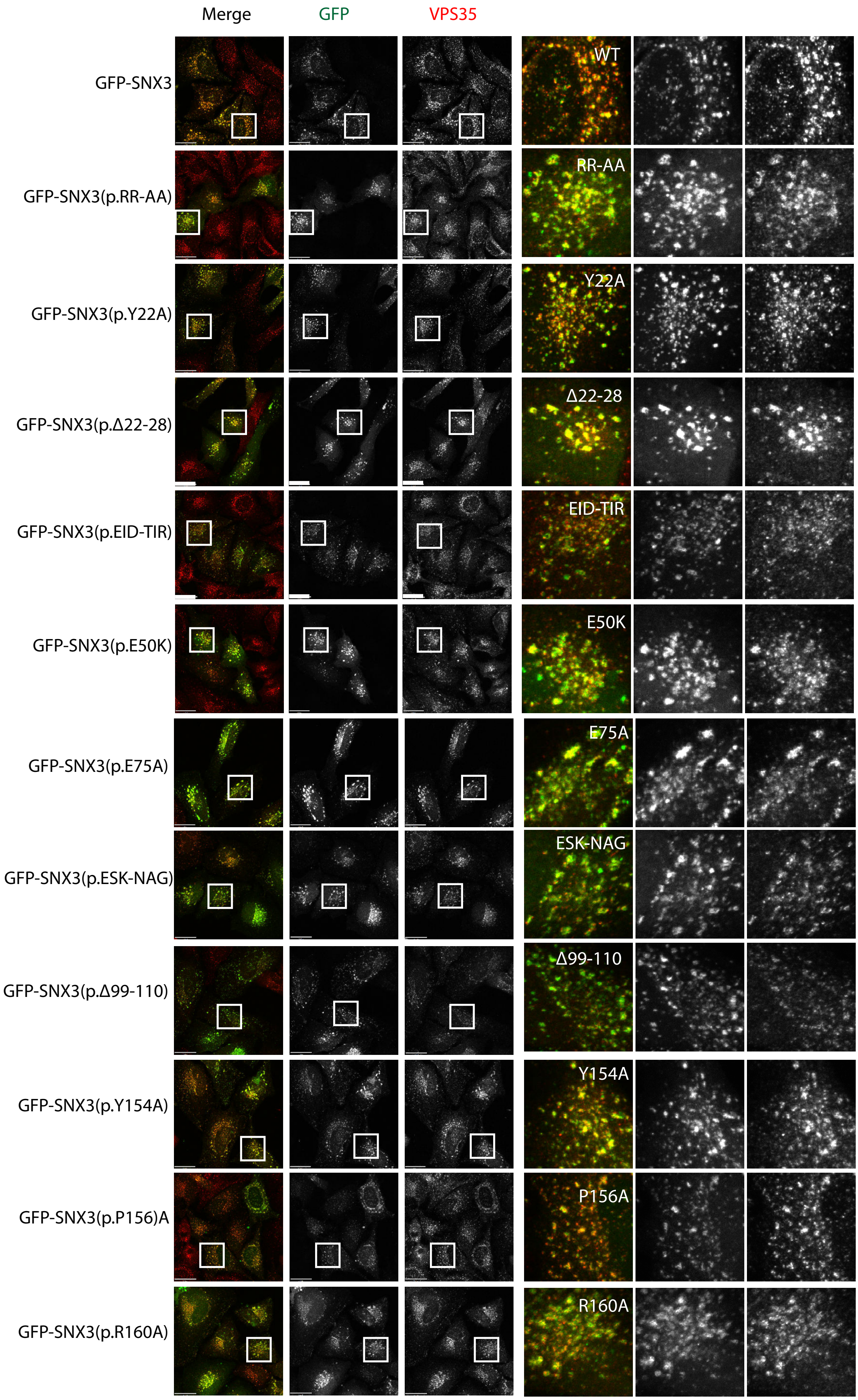

Supplementary figure 1: Mutant GFP-SNX3 constructs localise to endosomes.

Entire data set for Figure 1 C. Confocal images of HeLa cells transiently transfected to express wild type or mutated versions of GFP-SNX3 and subsequently stained for endogenous VPS26. Scale bar is 23 µm.

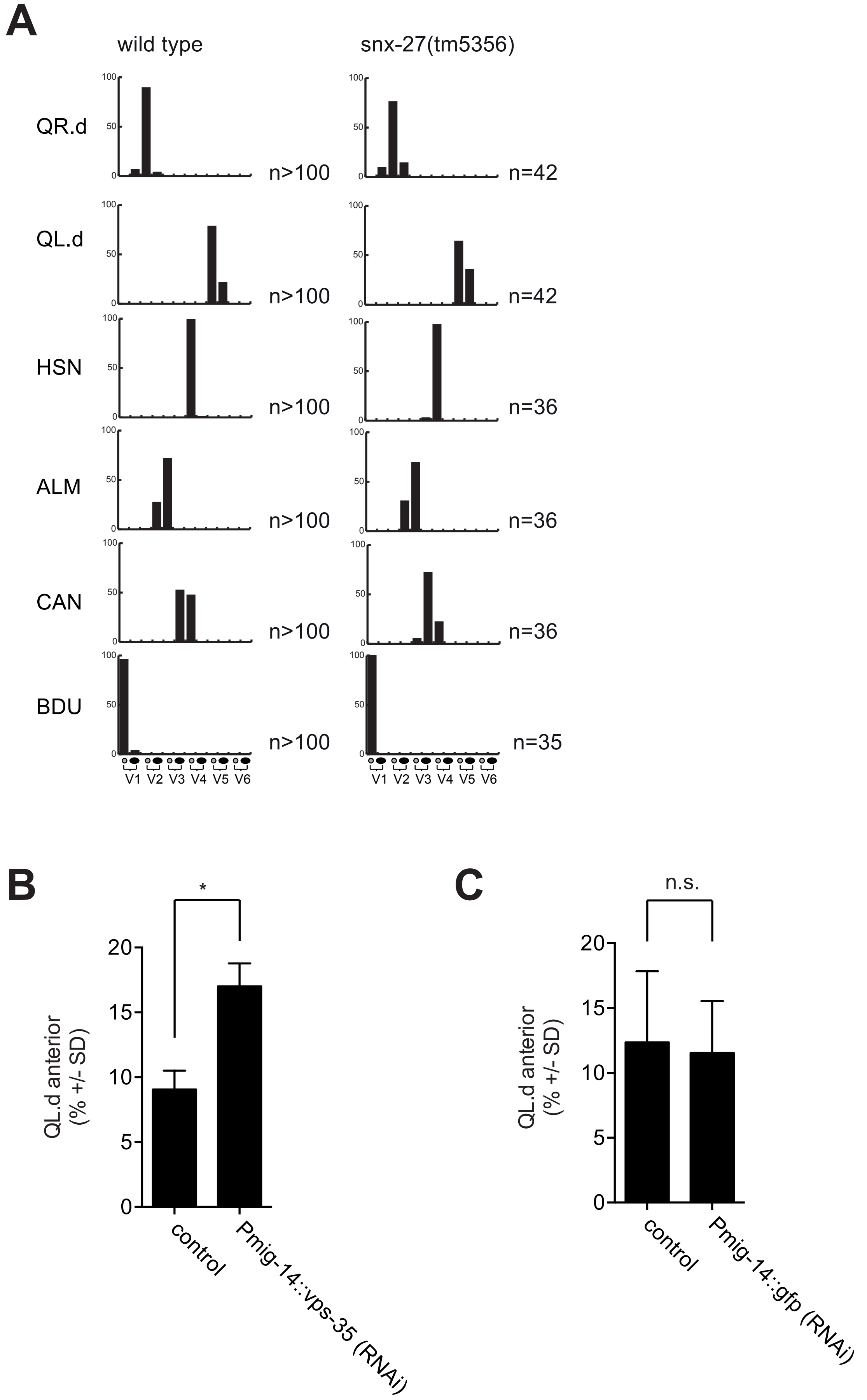

Supplementary figure 2.

(A) Loss of snx-27 does not affect the Wnt dependent cell migration of the HSN, ALM, CAN, BDU neurons and the Q neuroblasts. **(B)** Knock down of vps-35 in Wnt producing cells enhances the Wnt signaling phenotype (anterior localization of the QL.d) of vps-29 mutants. **(C)** Expression of gfp double stranded RNA has no effect on Wnt signaling in the vps-29 mutant background.

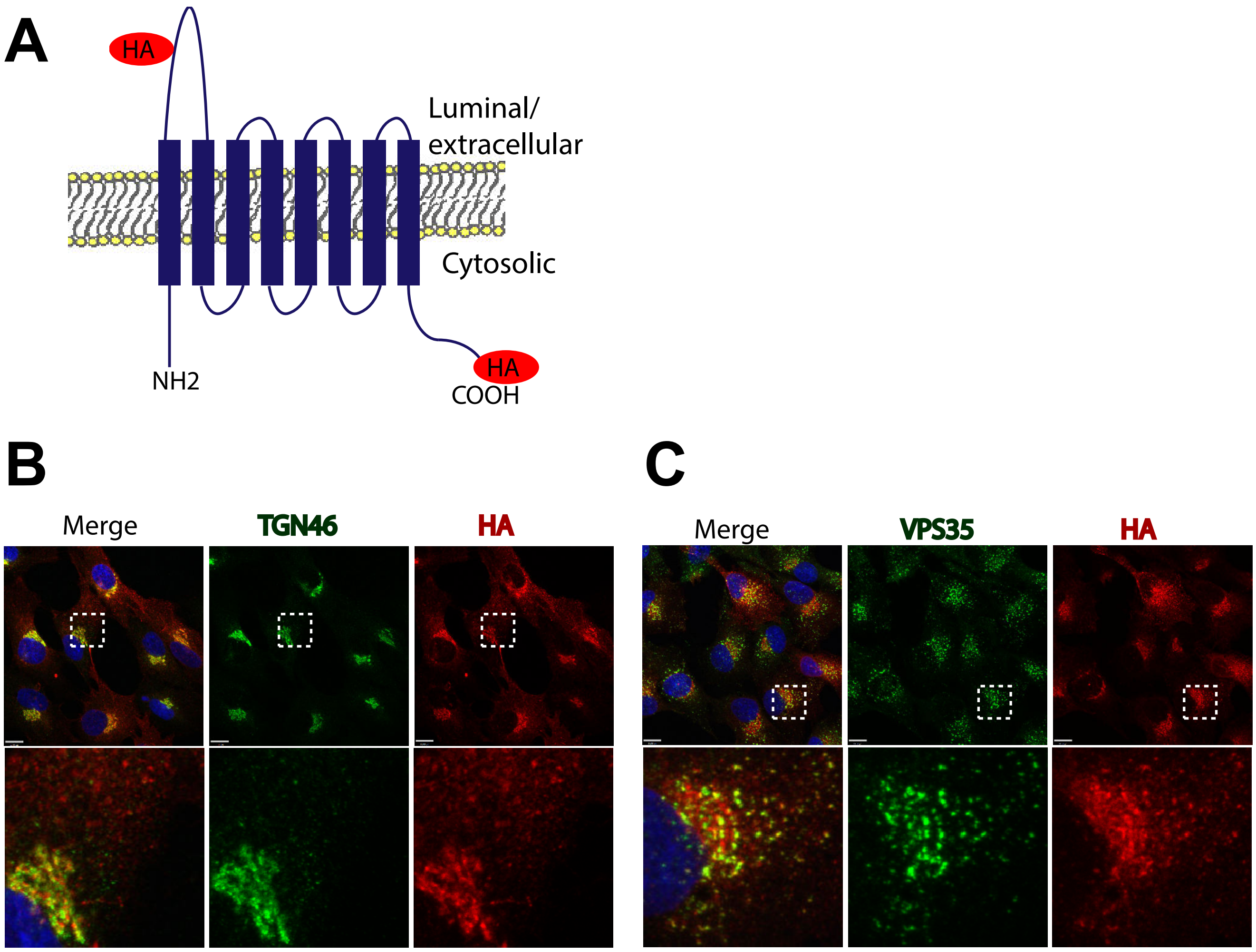

Supplementary Figure 3: HA-WLS localises to the trans-golgi network and VPS35-positive endosomes

(A) Schematic representation of the HA-WLS construct: two HA-tags are cloned onto Wntless, one on the luminal-facing and one on the cytosolic-facing side of the protein. **(B)** RPE-1 cells, stably expressing HA-WLS, were fixed and immuno-stained for HA and endogenous TGN46. The scale bar indicates 11 μm. **(C)** RPE-1 cells, stably expressing HA-WLS, were fixed and immuno-stained for HA and endogenous VPS35. The scale bar indicates 11 μm.

Supplementary Figure 4: Uncropped blots

FIgure 1B

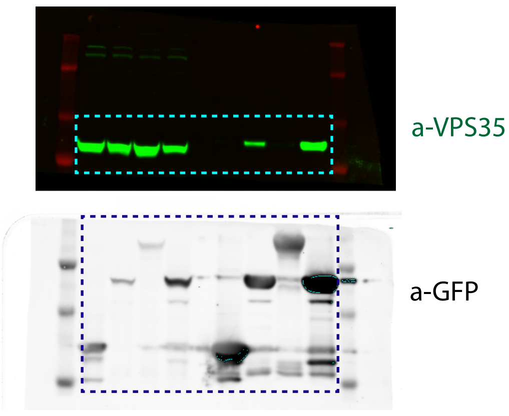

Figure 1D

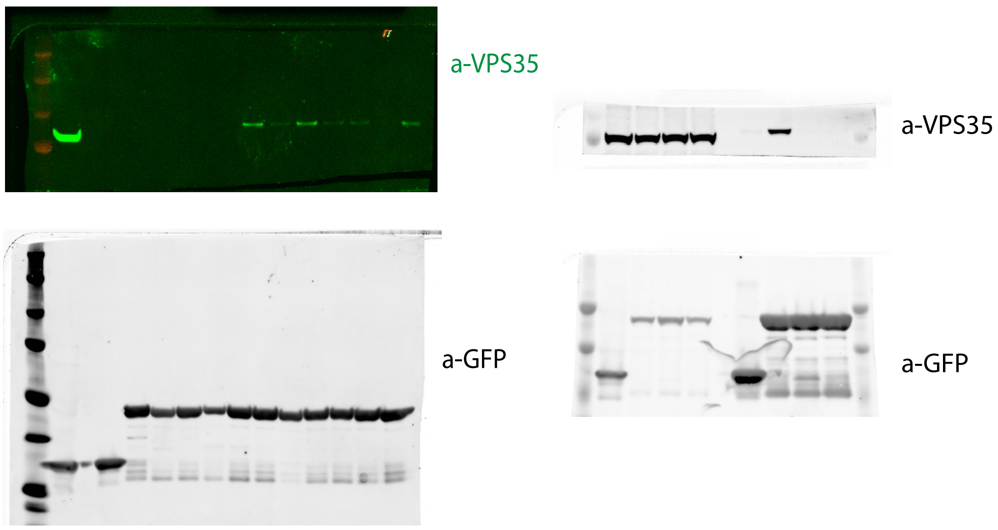

Figure 2D

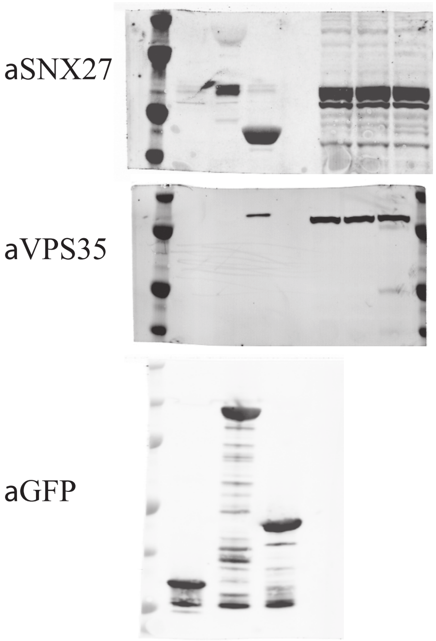

Figure 2E

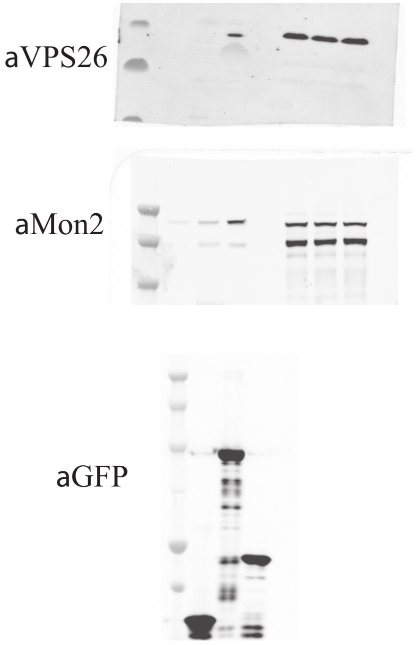

Figure 2F

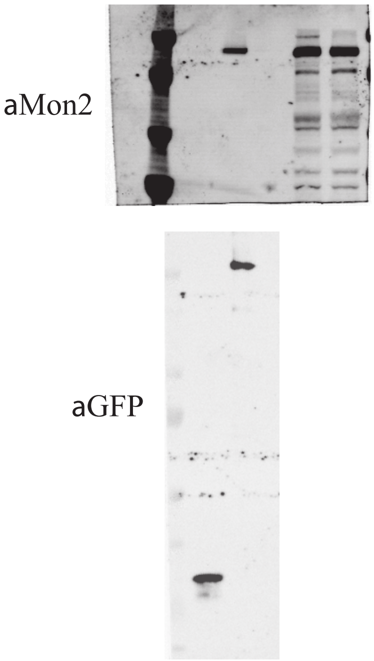

Figure 2G

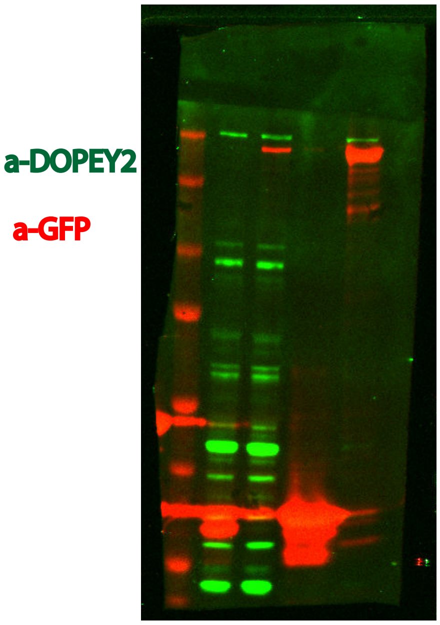

Figure 2H

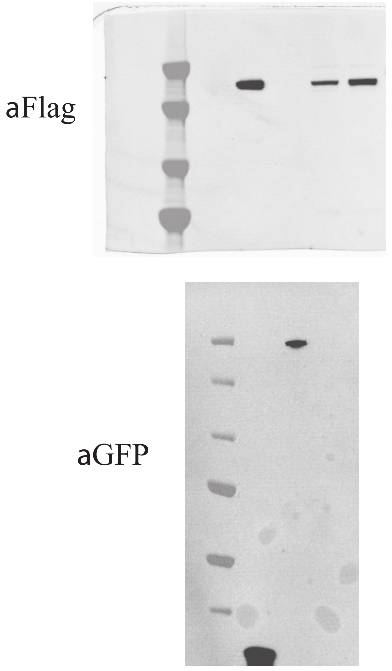

Figure 2I

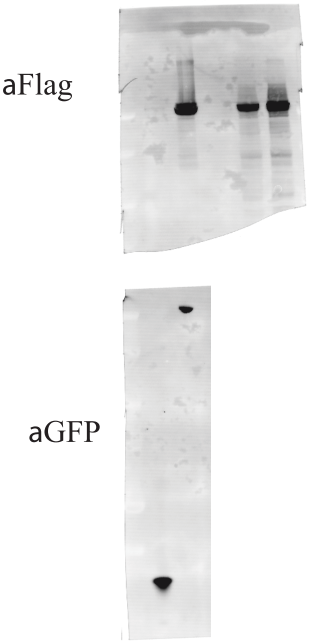

Supplementary Figure 4 (continued): Uncropped blots

Figure 2J

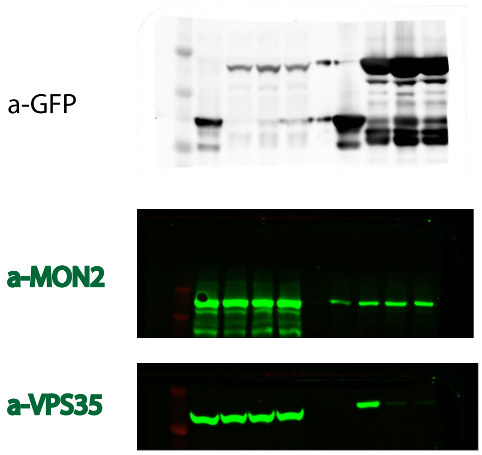

Figure 3A

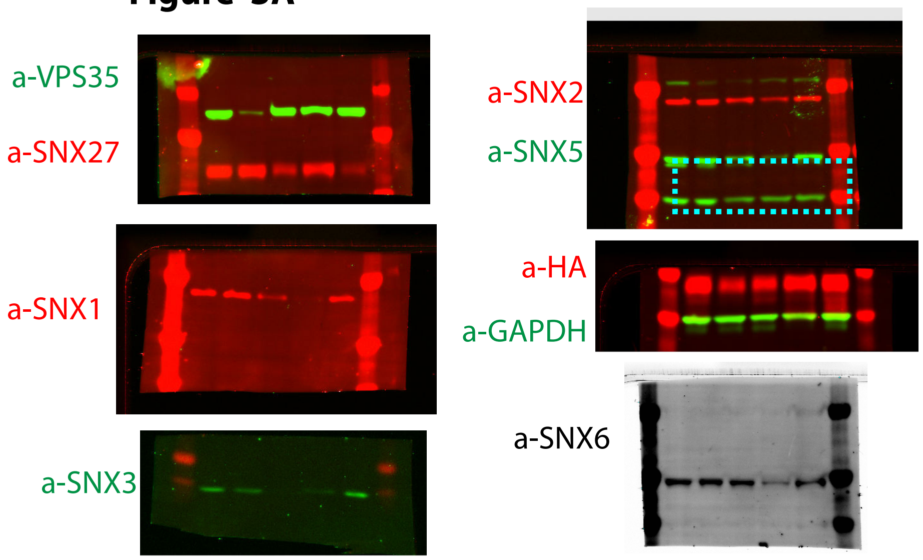

Figure 3B

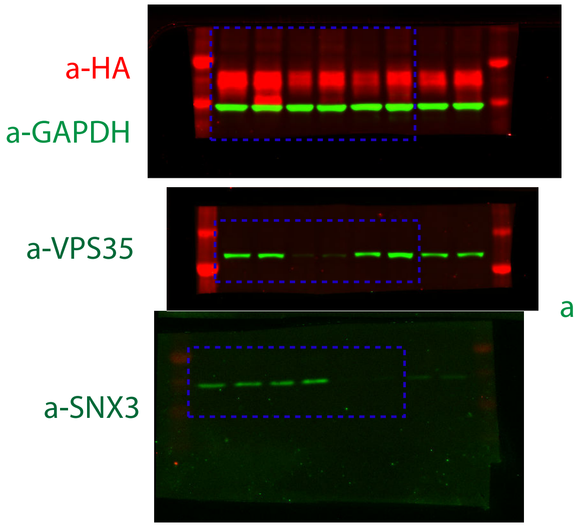

Figure 3C

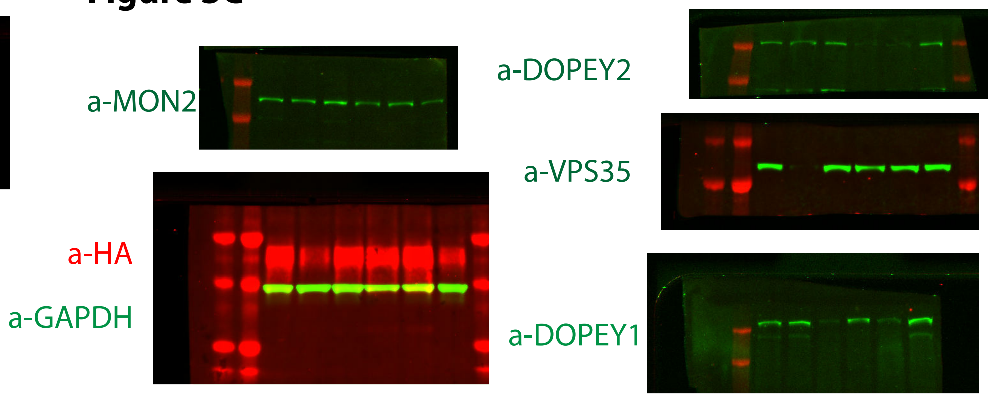

Figure 3D

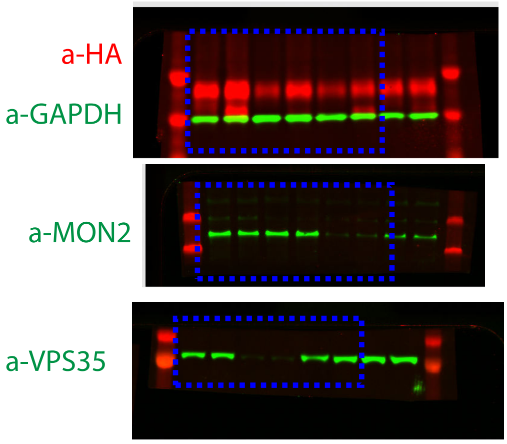

Figure 4H

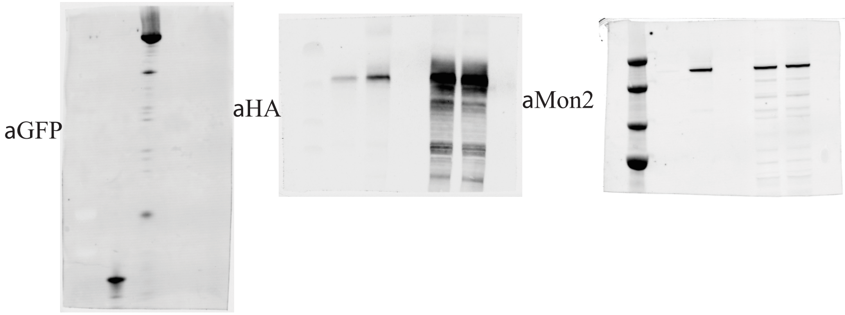

Figure 3E

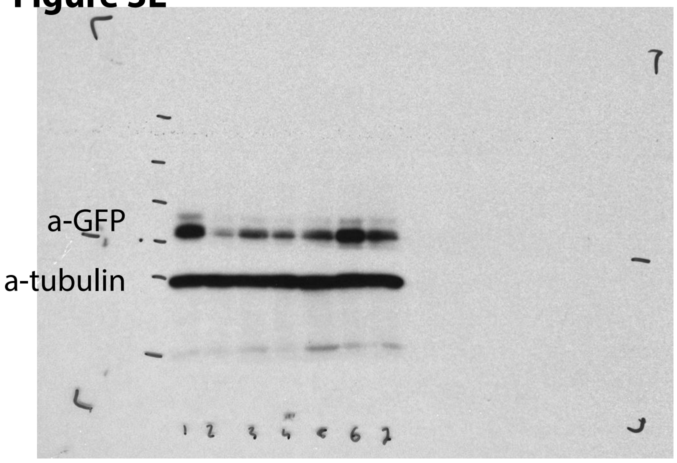

Figure 5A

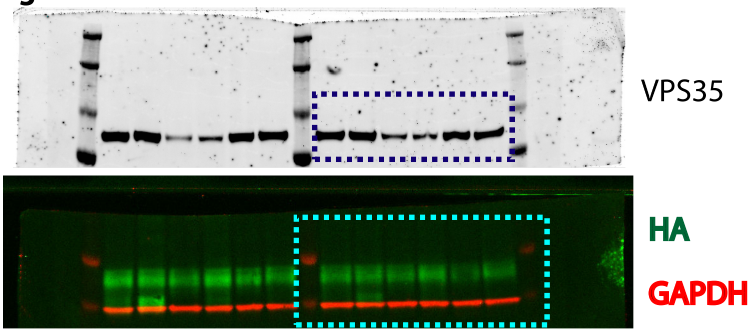

Supplement: Supplementary file 1 — Supplementary Information [file 41467_2018_6114_MOESM1_ESM.pdf]
