# Supplementary material for: SNX3-retromer requires an evolutionary conserved MON2:DOPEY2:ATP9A complex to mediate Wntless sorting and Wnt secretion
Source: Nat Commun. 2018 Sep 13;9:3737. doi: 10.1038/s41467-018-06114-3 (PMC6137200; doi:10.1038/s41467-018-06114-3)
Supplement: Supplementary file 2 — Description of Additional Supplementary Files [file 41467_2018_6114_MOESM2_ESM.pdf]

Description of Additional Supplementary Files

File Name: **Supplementary Data 1**
Description: **Unfiltered SILAC quantified interactome of GFP-SNX3 vs. GFP from human RPE-1 cells.** Human RPE-1 cells expressing either GFP or SNX3-GFP were cultured in light DMEM (GFP) or in heavy SILAC medium (GFP-SN3), followed by lysis and precipitation with GFP Nanotrap bead. After precipitation, beads were combined, bound proteins resolved by SDS-PAGE and subjected to detection and quantification of heavy and light peptides by LC-MS/MS.

File Name: **Supplementary Data 2**
Description: **Filtered SILAC quantified interactome GFP-SNX3 vs. GFP from human RPE-1 cells.** Human RPE-1 cells expressing either GFP or SNX3-GFP were cultured in light DMEM (GFP) or in heavy SILAC medium (GFP-SN3), followed by lysis and precipitation with GFP Nanotrap bead. After precipitation, beads were combined, bound proteins resolved by SDS-PAGE and subjected to detection and quantification of heavy and light peptides by LC-MS/MS. The resulting interactors were then thresholded to remove interactors with a Heavy/Light ratio less than 5 and with fewer than 2 peptides quantified. A network analysis was then performed to identify proteins known to interact with each other. Proteins known to interact with each other are colour coded the same colour.
